# Dramatic motor recovery following recanalization in bilateral pontine infarct with restricted diffusion

**DOI:** 10.1002/ccr3.3225

**Published:** 2020-08-09

**Authors:** Arnav Mahajan, Surasak Komonchan, Krida Na Songkhla, Anchulee Boontaworn, Supalak Nosai

**Affiliations:** ^1^ Department of Neurology Prasat Neurological Institute Bangkok Thailand; ^2^ Division of Neurology Lerdsin Hospital Bangkok Thailand; ^3^ Department of Neuroradiology Prasat Neurological Institute Bangkok Thailand

**Keywords:** acute basilar artery occlusion, endovascular treatment, lock in state, restricted diffusion

## Abstract

On findings of restricted diffusion in a bilateral pontine infarct, imaging solely may not predict a poor clinical outcome as a full motor recovery is possible. Hence, recanalization of an acutely occluded basilar artery should be carefully considered on a case by case basis.

## Graphical abstract

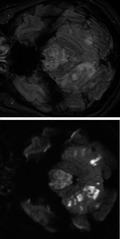

## 1 INTRODUCTION

The prognosis of bilateral pontine infarcts is known to be poor.^1^ The basilar artery is the sole arterial supply to both sides of the pons. If there is an acute basilar artery occlusion (BAO) that is not recanalized in time, a patient would likely be left with: quadriparesis; ophthalmoparesis; dysarthria; in the worse case scenario, lock‐in state.^1^ Patients will generally receive diffusion‐weighted imaging (DWI) and an apparent diffusion coefficient (ADC) score. If these detect an infarction in the brain, generally it suggests the affected areas are unsalvageable and irreversible. Currently, treatments using intravenous thrombolytic agents or intra‐arterial treatments for this condition remain unclear of its efficacy whereas mechanical thrombectomy (MT) is thought to be the most effective treatment.^2^ We report a case where a patient with BAO, whose brain magnetic resonance imaging (MRI) already showed evidence of restricted diffusion, received mechanical thrombectomy and had substantial improvement after a year—despite findings of an intense pontine infarction.

## 2 CASE PRESENTATION

**FIGURE 1:**
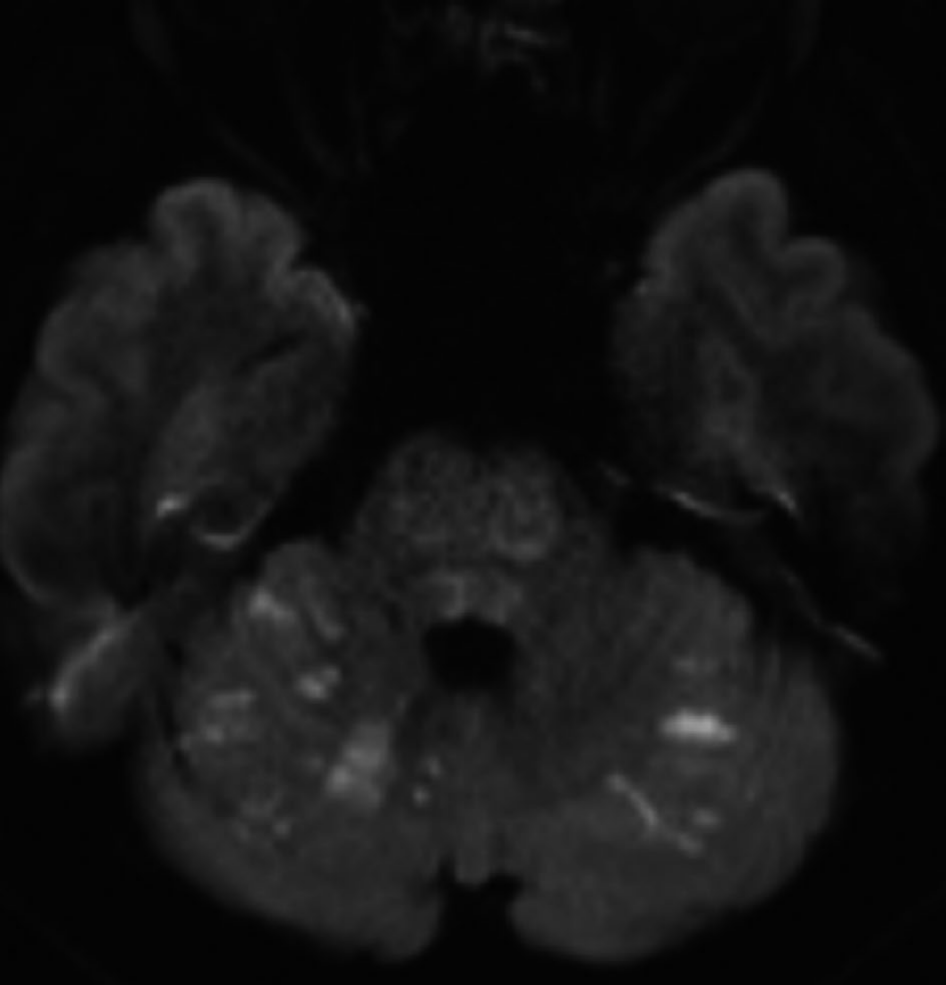
Axial DWI showing restricted diffusion of the pons and bilateral cerebellar hemispheres

**FIGURE 2:**
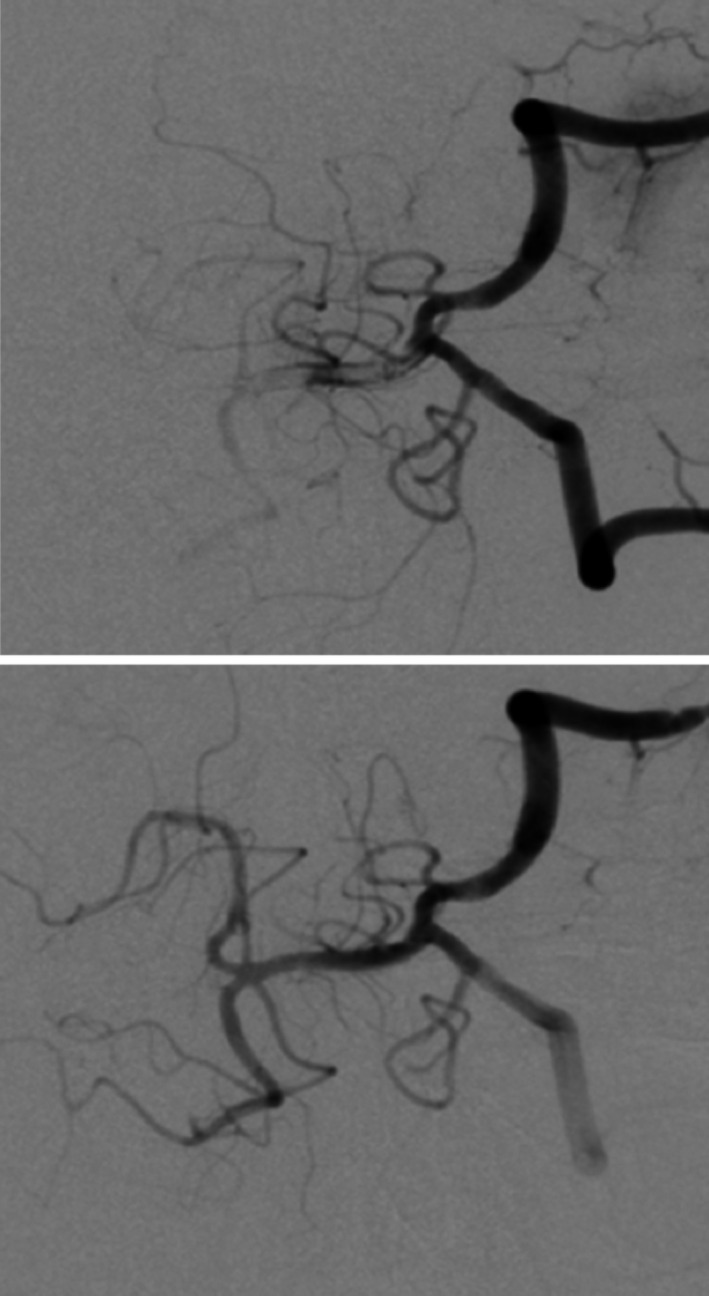
Pre‐ and post‐thrombectomy angiogram showing complete reperfusion of the basilar artery with normal antegrade flow

**FIGURE 3:**
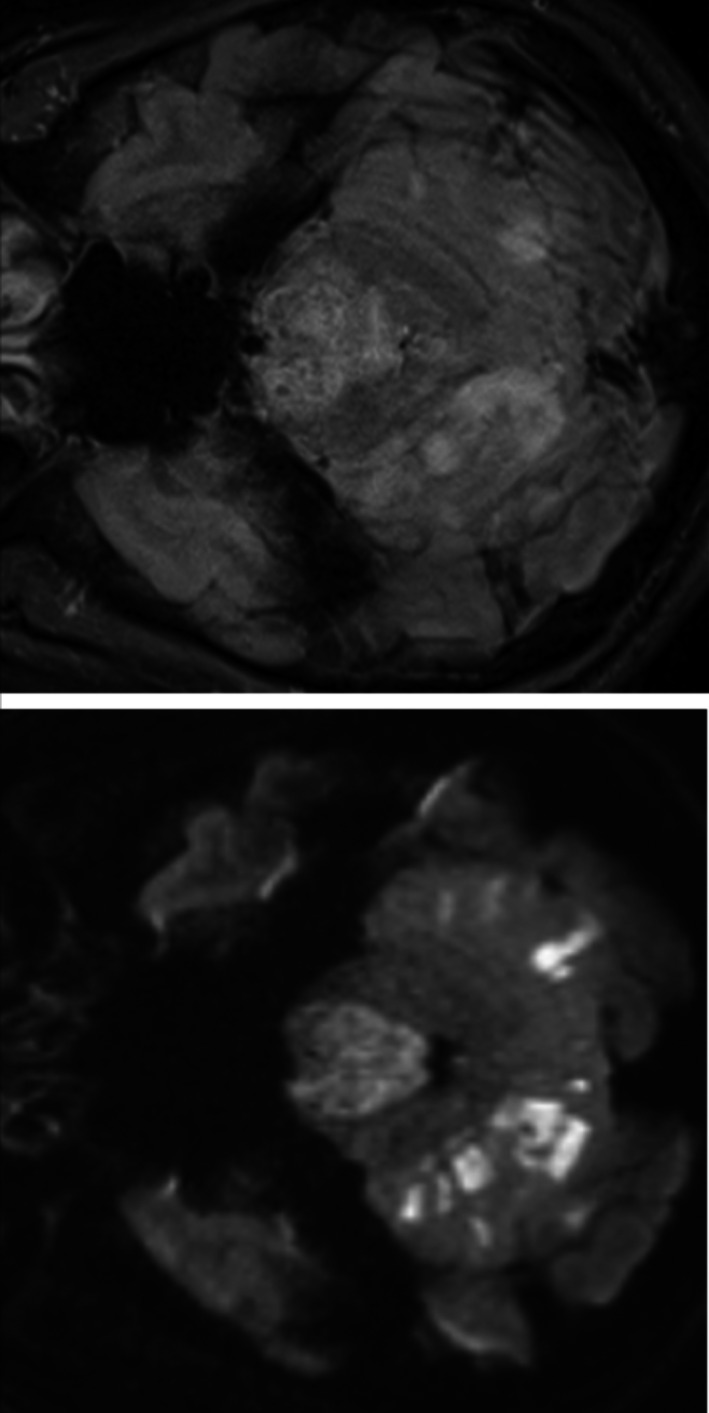
Twenty‐four hours post‐thrombectomy MRI: DWI (left) and fluid‐attenuated inversion recovery (FLAIR) (right) showing no increased area of pontine and cerebellar infarction, but more intense restricted diffusion with better defined lesion

A 47‐year‐old man was brought to a nearby hospital presenting with: sudden vertigo; vomiting; and gradual right‐sided hemiparesis that eventually evolved to quadriparesis, without any previous medical illness, which began a few hours earlier in the day. Computed tomography of his brain revealed no abnormal densities at the brainstem, and a computed tomography angiogram (CTA) of his brain revealed a proximal basilar artery occlusion. He was referred to our institution 11 hours after initial onset under the basis of being treated with mechanical thrombectomy. Upon arrival, he was intubated but was alert and keenly responsive. Neuroexamination showed left horizontal opthalmoplegia, a motor power grade of 2/5 on the left side, and hemiplegia on the right side that ultimately correlated to a National Institute of Health Stroke Scale score of 21. A brain MRI shortly after the examination demonstrated restricted diffusion at bilateral pons, with greater restriction on the left side, including a restricted diffusion at bilateral cerebellar hemispheres (Figure 1). Magnetic resonance angiography (MRA) of his brain demonstrated the same findings that were seen in the CTA. Following a discussion with his wife, we agreed to continue with endovascular treatment. After two attempts of stent retriever thrombectomy, complete recanalization was achieved (Figure 2). Antiplatelet therapy was applied for 24 hours after the procedure. The following day, an MRI and brain MRA follow‐up revealed hypersignal intensity at bilateral pons and cerebellar hemispheres that both appeared more distinctive than the initial MRI however remained limited to the same region (Figure 3). Electrocardiography monitoring and transthoracic echocardiogram were unremarkable. He was fed via nasogastric tube, received daily physical therapy, and was admitted in the stroke unit for 12 days before being referred for further rehabilitation at his previous hospital. He was discharged with an evaluated NIHSS score of 18.

Neuroexamination 1 month after his discharge revealed normal left horizontal opthalmoplegia and an improved left motor power grade of 4/5. However, neither his right side motor power nor his dysphagia had improved. At 1 year, a telephone and video call follow‐up had shown substantially improved swallowing and his wife showed evidence of him being ambulant. One‐year MRI follow‐up was not done since the patient lives far from our institution and had trouble with transportation. However, the patient was able to ambulate despite being previously being quadriplegic as a result of the infarct, which had shown no evidence of improvement after the MT was done—his ability to now live independently is diagnostic in its own right.

## 3 DISCUSSION

At many stroke centers across the world, acute BAO is commonly treated via endovascular therapy; however, it is currently inconclusive as to what method of treatment is the best. The current hypothesis of why this treatment works best on based upon early recanalization. The recanalization rate of endovascular treatment is far superior to that of intravenous thrombolytic drugs. This implicates the more aggressive treatment to achieve the best outcome. Despite the DWI and ADC that were positive in detecting restricted diffusion, the treatment provided to our patient overcame that general rule of the affected areas being irreversibly infarct. Only few reports have shown restricted diffusion of DWI and ADC at the pons being reversible—however, these reports did highlight the uncertainty of whether or not reversibility equates to a better clinical outcome.^3^ Unlike these reports, our case uniquely showed restricted diffusion of DWI and ADC at the pons before we proceeded with the mechanical thrombectomy—with DWI, ADC, and FLAIR at 24 hours showing an even more distinct infarct at the bilateral pons. Despite the extensively established infarct at the pons, his condition gradually improved until he was limitedly ambulant.

The amazing motor outcome of this bilateral pontine infarct could be explained by the reorganization of the unaffected corticospinal and corticobulbar tract (CST).^4^ Interestingly, the level of the pontine infarct can determine several characteristics of the motor outcome since different levels of the pons display different characteristics of the CST. At the lower pons, the compactness of the CST is higher than that of the upper pons—as it is measured in fractional anisotropy (FA)—despite the upper pons having a larger CST area.^5^ This suggests a linear neural structure of the CST that increases in density from upper to lower which could also clinically correlate to motor outcome.^5^ Upper pontine infarcts have been reported to have better clinical outcomes than patients with lower pontine infarcts.^6^ In our case, the patient had a middle pontine infarction. In a report by Kim et al, it suggests that middle pons infarctions led to severe hemiparesis whereas lesions of similar size in the paramedian rostral pons lead to dysarthria and clumsy hand syndrome (DA‐CH).^7^ This demonstrates that different presentation of symptoms occurs depending on the level of pontine infarct.^7^ The limitation of this report is that it is exclusively reporting single hemisphere pontine infarcts; however, we can make a correlation to our patient if we approach it as two single hemisphere pontine infarct cases rather than one bilateral pontine infarct. Our patient had a greater defined lesion on the left hand side which may be attributed to his severe right side hemiparesis that still lingered a month after recanalization; similarly, the lesser defined lesion on the right hand side may have led to his quicker left side motor recovery of 4/5 with persistent dysarthria. This supports both ideas of severe hemiparesis and DA‐CH occurring depending on the level of infarction. In a study by Zhang et al, findings demonstrate that the CST was associated with motor function recovery after 90 days of pontine infarction, but after 180 days, the CST no longer associated with such recovery, which suggests that recovery even after severe pontine infarcts could result in motor redevelopment.^8^ We can easily see this on our patient where in the less severe right side lesion, recovery was achieved before 90 days and it may be attributed to CST reorganization; however, on the more severe left side lesion,the patient only recovered full motor function after a year which may suggest that the CST was not involved in recovery anymore. Multiple instances of motor pathway salvaging were found which has also been reported on many occasions.^8^, ^9^, ^10^, ^11^ Suggested mechanisms of this include the peri‐infarct CST taking over all motor function or even the lateral CST dominating major movement.^9^, ^10^, ^11^ We suggest that it was the peri‐infarct CST that assisted in motor recovery of the right side lesion, since if the lateral CST was to assist in recovery then it would also be involved as the peri‐infarct CST of the left side lesion. However, since there was limited recovery a month after recanalization with regards to the right side motor movements, we refute this claim. Additionally, since he did not have any previous medical illness and was young, those factors too might have contributed to his recovery. We understand that conducting diffusion tensor imaging (DTI) would have been highly diagnostic and would have provided substantial evidence about the motor recovery of the patient; however, he had refused to come in for a follow‐up MRI.

After the MT, his basilar artery had appeared normal without any remaining stenosis. This leads us to suspect that the cause of the stroke was likely from emboli of undetermined origins. Cumulatively, with other reports that indicated restricted diffusion findings in a bilateral pontine infarct, imaging solely may or may not predict a poor clinical outcome; hence, recanalization of an acutely occluded basilar artery should be carefully considered.^12^, ^13^ More studies are necessary to determine whether recanalization with endovascular treatment or intravenous thrombolysis has a role in this situation.

## CONFLICT OF INTEREST

None.

## AUTHOR CONTRIBUTIONS

AM: wrote the manuscript and reviewed the literature. SK: mentored to formulate core understanding, gave suggestions in manuscript development, and was involved in the care of the patient. KnS: edited the manuscript and reviewed the literature. AB: edited the manuscript and was involved in the care of the patient. SN: edited the manuscript and assisted in radiographic imaging used in this patient.
